# Disparate Metabolomic Responses to Fructose Consumption between Different Mouse Strains and the Role of Gut Microbiota

**DOI:** 10.3390/metabo11060342

**Published:** 2021-05-26

**Authors:** In-Sook Ahn, Justin Yoon, Graciel Diamante, Peter Cohn, Cholsoon Jang, Xia Yang

**Affiliations:** 1 Department of Integrative Biology and Physiology, University of California, Los Angeles, CA 90095, USA; iahn@ucla.edu (I.-S.A.); jmyoon0405@g.ucla.edu (J.Y.); gdiam001@ucla.edu (G.D.); peter@cohnkin.com (P.C.); 2 Department of Biological Chemistry, University of California, Irvine, CA 92697, USA; choljang@uci.edu; 3 Brain Research Institute, University of California, Los Angeles, CA 90095, USA; 4 Molecular Biology Institute, University of California, Los Angeles, CA 90095, USA; 5 Institute for Quantitative and Computational Biosciences, University of California, Los Angeles, CA 90095, USA

**Keywords:** fructose, metabolomics, gut microbiota, genetic background, personalized disease risk, metabolic syndrome

## Abstract

High fructose consumption has been linked to metabolic syndrome, yet the fructose-induced phenotypes, gene expression, and gut microbiota alterations are distinct between mouse strains. In this study, we aim to investigate how fructose consumption shapes the metabolomic profiles of mice with different genetic background and microbiome. We used fructose-sensitive DBA/2J (DBA) and fructose-resistant C57BL/6J (B6) mice given 8% fructose or regular water for 12 weeks. Plasma and fecal metabolites were profiled using a liquid chromatography-tandem mass spectrometry based global metabolomic approach. We found that the baseline metabolomic profiles were different between DBA and B6 mice, particularly plasma metabolites involved in lipid metabolism and fecal metabolites related to dipeptide/amino acid metabolism. In response to fructose, DBA mice showed a distinct decrease of plasma branched chain fatty acids with concordantly increased branched chain amino acids, which were correlated with adiposity; B6 mice had significantly increased plasma cholesterol and total bile acids, accompanied by decreased fecal levels of farnesoid X receptor antagonist tauro-β-muricholate, which were correlated with fructose-responsive bacteria *Dehalobacterium*, *Magibacteriaceae*, and/or *Akkermansia*. Our results demonstrate that baseline metabolomic profiles differ and respond differentially to fructose between mice with different genetic background and gut microbiota, which may play a role in individualized risks to fructose-induced metabolic syndrome.

## 1. Introduction

High fructose corn syrup is one of the common dietary components of the western diet, and sugar-sweetened beverages are the major contributor to dietary fructose intake in the US [1]. Increased high fructose consumption parallels a rise in the prevalence of metabolic syndrome, obesity, type 2 diabetes, and cardiovascular disease. Multiple lines of evidence support that physiological responses to diets are highly context-dependent: genetic background, metabolic state, physical activity, and gut microbiome can all contribute to individualized response to diets such as high fructose diets [2,3]. Previous mouse studies have demonstrated that high fructose consumption results in strain-specific responses in metabolic phenotypes [4,5], tissue-specific transcriptomic profiles [6], and gut microbiota composition [7], supporting that fructose response is context dependent. Individualized response to fructose has also been observed in human populations [8,9].

Metabolomics measures biochemical phenotypes which reflect genetic contribution, gut microbiome activity, and lifestyle choices such as smoking, exercise, and diet, thus providing highly integrated information of biological status [10,11]. Application of metabolomics has led to the identification of biomarkers that are associated with disease risks, prediction of disease development, and intervention outcomes. Metabolites are produced endogenously by cells or derived from the environment or diets. The gut microbiota is also a critical component in shaping metabolite profiles through its metabolism distinct from the host [12]. There are about 500 to 1000 species of gut microorganisms in adult humans, which break down and metabolize complex nutrients to produce numerous metabolites. Metabolites, in turn, affects the host and the gut microbiota to influence metabolic homeostasis and physiology. Various bioactive metabolites from diet, microbiota, and host metabolism have been associated with metabolic disorders or disease risk. For instance, branched chain amino acids (BCAAs) including leucine, isoleucine and valine are essential amino acids that must be obtained from food. These amino acids are readily catabolized by gut microbiota or host metabolism to produce metabolites such as α-ketoacids or branched chain fatty acids (BCFAs) [13,14]. Elevated levels of BCAAs and decreased BCFAs have been associated with the development of obesity-associated insulin resistance [11,15,16]. As another example, short-chain fatty acids (SCFAs) including acetate, butyrate, and propionate, are major metabolites produced by bacterial fermentation of dietary fiber or amino acid substrates [14], maintain host energy homeostasis [17] and protect from gut dysbiosis [12,18,19]. 

Fructose consumption is also known to induce alterations in numerous metabolites. Fructose is absorbed in the small intestine and metabolized to glucose, glycerate, and other organic acids. When intestinal fructose absorption is incomplete at high fructose consumption, the unabsorbed fructose reaches the colonic microbiota and is used to generate SCFAs [20]. High fructose consumption also alters metabolites including acylcarnitine, diacylglycerol, and uric acid [21,22], which are related with metabolic disorders. However, the role of metabolites in differential fructose response between genetic backgrounds remains unclear.

We have previously reported that genetically distinct C57BL/6J (B6) and DBA/2J (DBA) mice demonstrated significant differences in their transcriptome, gut microbiome, and metabolic phenotypes upon 8% fructose water consumption for 12 weeks [6,7]. DBA mice had significantly increased adiposity and glucose intolerance in response to fructose, whereas B6 mice had increased cholesterol profiles. Additionally, fructose altered different sets of microbial taxa between mouse strains, including *Akkermansia*, *Dehalobacterium*, and unknown genus of *Mogibacteriaceae* in B6, and unknown genus of *Rikenellaceae*, *Pseudomonadaceae*, *Pseudomonas* in DBA mice. These bacteria altered by fructose demonstrated mouse strain-specific correlations with host transcriptome and metabolic phenotypes. Since we observed similar fructose and caloric intake in B6 and DBA, this suggests that overall energy intake and fructose intake do not explain the differential metabolic responses to fructose between mouse strains [6,7]. 

**Figure 1:**
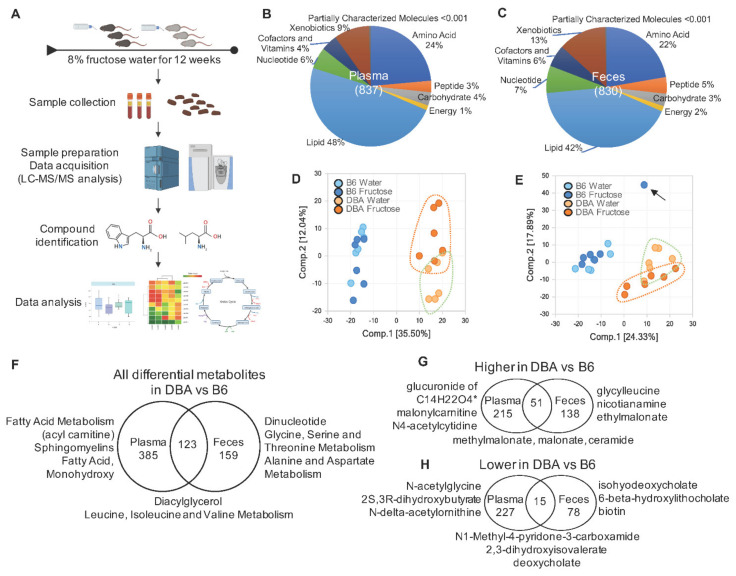
Metabolomic profiles in plasma and feces of DBA and B6 mice. (**A**) Schematic study design. (**B**,**C**) Distribution of plasma (**B**) and fecal (**C**) metabolites within nine major metabolic pathways. The numbers shown in parentheses are the total metabolites we have identified in plasma (**B**) and feces (**C**). (**D**,**E**) Principal component analysis (PCA) plot assessing the variance of metabolites between B6 water group, B6 fructose group, DBA water group, and DBA fructose group in the plasma (**D**) and feces (**E**). The percentages of variance explained by the first (Comp. 1) and second (Comp. 2) principal components are shown in brackets. The arrow in (**E**) indicates an outlier detected, which was removed from subsequent analyses. (**F**–**H**) Venn diagrams showing the numbers of all differential (**F**), higher (**G**), or lower (**H**) metabolites in DBA compared with B6 without fructose consumption. We further compared these differential metabolites between plasma and feces to infer differential metabolites potentially originated from the gut microbiota. Top representative pathways are shown in (**F**), which are similar for the higher and lower metabolites in (**G**,**H**). Therefore, top differential metabolites instead of pathways were shown in (**G**,**H**). *n* = 5–6/group/mouse strain. Differentially abundant baseline metabolites in DBA compared with B6 were determined by ANOVA contrasts (*q* < 0.05). Symbol (*) next to metabolite indicates that the compounds have not been officially confirmed based on a standard, but that we are confident in its identity.

To further investigate the interactions of fructose intake, genetic background, gut microbiota, and metabolome, in this study we characterized the global metabolite profiles of DBA and B6 mice. Using a subset of available plasma and fecal samples from the same animals as our previous studies in [6,7], we used a liquid chromatography-tandem mass spectrometry (LC-MS/MS)-based global metabolomic approach and revealed significant differences in the metabolite profiles with or without fructose consumption as well as between DBA and B6 mice (Figure 1A). Next, we identified mouse strain-specific metabolites and pathways that associate with fructose response, gut microbiota, and host metabolic phenotypes.

## 2. Results

### 2.1. Disparate Metabolomic Profiles between DBA and B6 Mice

From both B6 and DBA mice, 837 and 830 metabolites were identified in plasma and feces, respectively. Among the identified metabolites, nearly half belonged to the lipid pathway in both plasma (48%) and feces (42%), followed by amino acids, xenobiotics, nucleotides, cofactor and vitamins, peptides, carbohydrates, and energy pathways (Figure 1B,C). Principle component analysis (PCA) revealed dramatically distinct clustering between B6 and DBA samples for both plasma (Figure 1D) and fecal (Figure 1E) metabolite profiles. Intriguingly, DBA samples were separated by fructose treatment for both plasma and fecal metabolite profiles, while B6 samples did not show distinct separation by fructose consumption.

We next examined the difference in baseline metabolite profiles between DBA and B6 mice without fructose consumption. By ANOVA contrasts at *q* < 0.05 after multiple testing correction, 508 and 282 metabolites were differentially abundant between DBA and B6 mice in plasma and feces, respectively. Among them, 266 and 189 metabolites were higher, while 242 and 93 metabolites were lower in plasma and feces of DBA compared with B6 mice, respectively (Figure 1F–H, Table S1). The plasma metabolome reflects the collective outcome of host/gut microbiota metabolism. To identify plasma metabolites that were potentially derived from the gut microbiota, we compared the differential metabolites in plasma with the ones in feces. Among the 508 differential metabolites in plasma, 123 overlapped with fecal differential metabolites, with 51 higher and 15 lower in both plasma and feces of DBA compared to B6. We speculate that some of these concordant differential metabolites were derived from gut microbiota (Figure 1F–H, Table S1). Pathway enrichment analysis revealed that the plasma differential metabolites between mouse strains were associated with lipid metabolism including acyl carnitine (long chain saturated), sphingomyelins, and diacylglycerol, indicating that fatty acid metabolism is the major disparate metabolic pathway between DBA and B6 mice in plasma. In feces, the top representative pathways for the differential metabolites are associated with dipeptide metabolism, amino acids metabolism, and endocannabinoid. Pathways related to diacylglycerol and BCAA metabolism were significantly different between DBA and B6 in both plasma and feces (Figure 1F, Table S2). 

### 2.2. Fructose-Responsive Metabolites in DBA Mice

**Figure 2:**
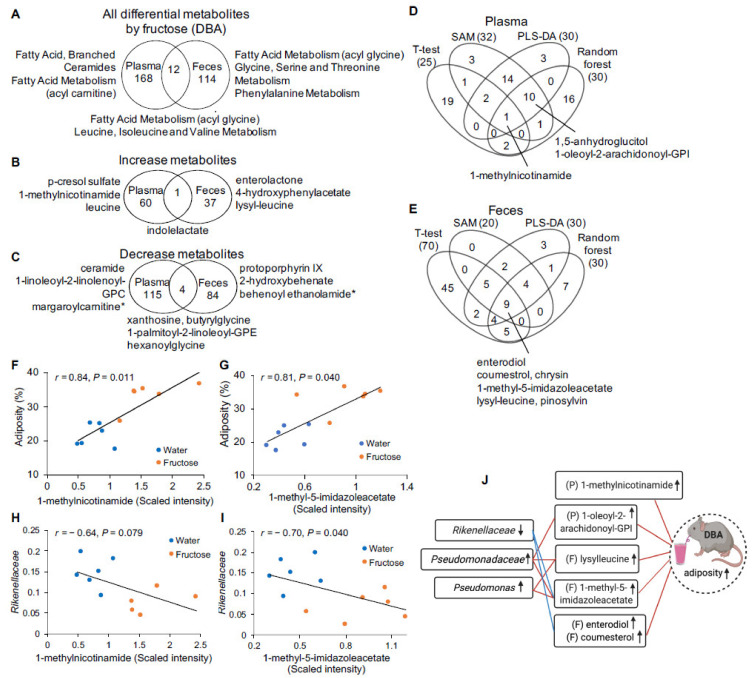
Fructose-responsive metabolites in plasma and feces of DBA mice. (**A**) Venn diagram showing the number of fructose-responsive metabolites and the representative pathways that were overlapping or unique between plasma and feces in DBA mice. (**B**,**C**) Venn diagram showing the number of the increased (**B**) and decreased (**C**) metabolites by fructose and the top metabolites that were overlapping or unique between plasma and feces in DBA mice. (**D**,**E**) Venn diagrams representing overlapping significant metabolites altered by fructose in plasma (**D**) and feces (**E**) across four statistical methods: statistical significance (*t*-test/fold change), Significance Analysis of Metabolites (SAM), Partial-least squares discriminant analysis (PLS-DA), and Random forest. (**F**–**I**) Correlation plots of metabolites with the adiposity phenotype (**F**,**G**) and fructose-responsive microbiota (proportion, 0–1) (**H**,**I**) in DBA mice with and without fructose treatment. r, Biweight midcorrelation (bicor) coefficient; *P*, Benjamini-Hochberg adjusted *p*-values (FDR). (**J**) Summary of the correlation between fructose-responsive microbiota, metabolites, and metabolic phenotype (adiposity) of DBA mice. Increased or decreased levels of microbiota, metabolites, and metabolic phenotype (adiposity) upon fructose consumption were indicated by up or down arrows, respectively. P, plasma; F, feces. *n* = 5–6/group/strain. Fructose-responsive metabolites were determined by ANOVA contrasts (*p* < 0.05). Symbol (*) next to metabolite indicates that the compounds have not been officially confirmed based on a standard, but that we are confident in its identity.

In DBA mice, we identified 50 plasma and 20 fecal metabolites that were altered by fructose consumption at *q* < 0.05 by ANOVA contrasts, with no overlapping metabolites between plasma and feces. The small number of metabolites identified using the stringent cutoff limited downstream pathway analysis, therefore for pathway analysis, we used a *p* < 0.05 as the threshold. Using this cutoff, 180 plasma metabolites were altered by fructose, with 61 metabolites increased and 119 decreased; in feces, we detected 126 metabolites altered by fructose, with 38 metabolites increased and 88 decreased (Figure 2A–C, Table S1). Twelve plasma metabolites including increased indolelactate, and decreased xanthosine, butyrylglycine, hexanoylglycine, and 1-palmitoyl-2-linoleoyl-glycero-3-phosphoethanolamine (GPE) were overlapped with fecal metabolites, suggesting that some of them were potentially derived from gut microbiota metabolism. Pathway enrichment analysis revealed that fructose significantly perturbed 11 metabolic pathways in plasma, including fatty acid, branched (BCFA), ceramides, fatty acid metabolism (acyl carnitine), and nicotinate and nicotinamide metabolism. In feces, fructose affected six metabolic pathways including fatty acid metabolism (acyl glycine), serine-threonine metabolism, and phenylalanine metabolism. Two pathways, fatty acid metabolism (acyl glycine) and BCAA metabolism, were altered in both plasma and feces by fructose consumption (Figure 2A, Table S2). 

To prioritize metabolites altered by fructose consumption in DBA mice, we highlighted those which showed statistical robustness across various analytical methods. We analyzed global metabolomics data using four different statistical and machine learning algorithms: *t*-test, Significance Analysis of Metabolites (SAM), Partial-least squares discriminant analysis (PLS-DA), and Random forest. In plasma, 1-methylnicotinamide, which is generated from nicotinamide (vitamin B3) and reported to increase in obesity, diabetes, and coronary artery disease [23,24], was identified across all four statistical tests. In feces, nine metabolites were detected across all four methods, which include 1-methyl-5-imidazoleacetate, lysyl-leucine (a dipeptide of lysine and leucine), and diet-derived polyphenol metabolites such as enterodiol and coumestrol (Figure 2D,E, Table S3). These metabolites represent robust fructose-responsive metabolites in DBA mice which showed obesity and glucose intolerance. However, we note that statistical robustness does not imply higher biological importance, and other differential metabolites shown in Table S3 are important to consider as well.

### 2.3. Correlation of Fructose-Responsive Metabolites with Metabolic Phenotypes or Fecal Microbiota in DBA Mice

To evaluate the relationship between metabolites and metabolic phenotypes, we next performed correlation analysis between fructose-responsive metabolites and metabolic phenotypes collected from the same sets of DBA mice, since significant obesity development and glucose intolerance were observed upon fructose consumption in this mouse strain [6]. Thirty-eight metabolites in plasma and 10 metabolites in feces were correlated with one or more of the metabolic phenotypes, and over 80% of them were correlated with adiposity (Table S4). Multiple metabolites in plasma and feces that were prioritized in Figure 2D,E were also correlated with adiposity (Figure 2F,G, Table S4). For example,1-methylnicotinamide, which was increased by 2.13 folds by fructose in plasma, was positively correlated with body weight and adiposity, and negatively correlated with lean mass and free fatty acid. 1-methyl-5-imidazoleacetate and lysyl-leucine in feces were increased by fructose treatment and correlated with adiposity. 

Next, to explore the relationship between metabolites and gut microbiota, which may contribute to the fructose response, we performed correlation analysis between fructose-responsive metabolites and fructose-responsive fecal microbiota in DBA mice, including the unknown genus of *Rikenellaceae* which was decreased, and *Pseudomonadaceae* and *Pseudomonas*, which were increased upon fructose treatment [7]. Two plasma metabolites, 1,5-anhydroglucitol and 1-oleoyl-2-arachidonoyl-GPI were correlated with the unknown genus of *Rikenellaceae* and unknown genus of *Pseudomonadaceae*, respectively (Table 1). Nineteen fecal metabolites were correlated with one or more of the three fructose-responsive bacteria. Among them, 1-methyl-5-imidazoleacetate was correlated with all three bacteria. Lysyl-leucine was positively correlated with unknown genus of *Pseudomonadace* and *Pseudomonas*. Coumestrol and enterodiol were negatively correlated with unknown genus of *Rikenellaceae*. We also noted that two endocannabinoids, behenoyl ethanolamide and lignoceroyl ethanolamide, which were decreased by fructose, showed correlation with all three fructose-responsive bacteria (Table 1, Figure 2H,I). This finding was particularly intriguing because endocannabinoids are endogenous lipid-based neurotransmitters that regulate feeding behaviors and lipid metabolism [25]. Taken together, across metabolites, gut bacteria, and phenotypes, most of the prioritized metabolites in Figure 2D,E were correlated with bacteria and/or adiposity (Figure 2J), which suggests that these metabolites have a strong relationship with the microbiota and may play a role in fructose-induced obesity in DBA mice.

### 2.4. Fructose-Responsive Metabolites in B6 Mice

**Figure 3:**
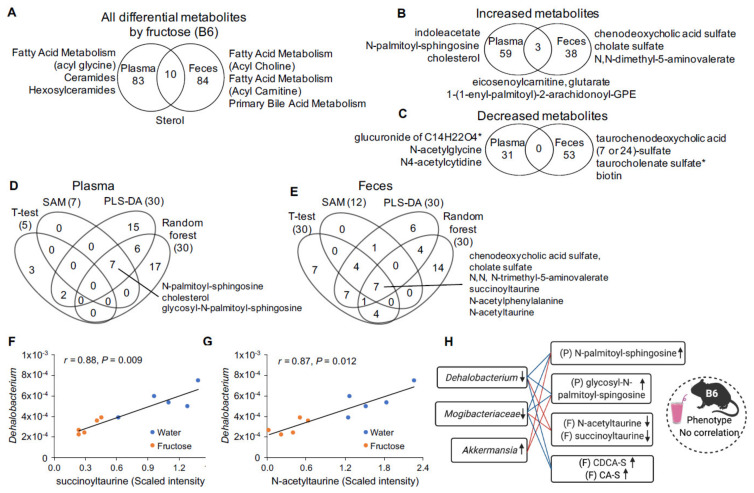
Fructose-responsive metabolites in plasma and feces of B6 mice. (**A**) Venn diagram showing the number of fructose-responsive metabolites and the representative top pathways that were overlapping or unique between plasma and feces of B6 mice. (**B**,**C**) Venn diagrams showing the number of the increased (**B**) and decreased (**C**) metabolites by fructose and the top metabolites that were overlapping or unique between plasma and feces of B6 mice. (**D**,**E**) Venn diagrams representing overlapping significant metabolites altered by fructose in plasma (**D**) and feces (**E**) across four statistical methods: statistical significance level (*t*-test/fold change), Significance analysis of metabolites (SAM), Partial-least squares discriminant analysis (PLS-DA), and Random forest. (**F**,**G**) Correlation plots between metabolites and fructose-responsive microbiota (proportion, 0–1) of B6 mice with or without fructose treatment. r, Biweight midcorrelation (bicor) coefficient; *P*, Benjamini-Hochberg adjusted *p*-values. (**H**) Summary of the correlative relationships between fructose-responsive microbiota, metabolites, and metabolic phenotype of B6 mice. Increased or decreased microbiota, metabolites, and metabolic phenotype upon fructose consumption were indicated by up or down arrows, respectively. CDCA-S, chenodeoxycholic acid sulfate; CA-S, cholate sulfate; P, plasma; F, feces. *n* = 5–6/group/strain. Fructose-responsive metabolites were determined by ANOVA contrasts (*p* < 0.05). Symbol (*) next to metabolites indicates that the compounds have not been officially confirmed based on a standard, but confident in its identity.

In B6 mice, we identified 8 plasma and 35 fecal metabolites altered by fructose at *q* < 0.05 by ANOVA contrasts, with two overlapping metabolites, campesterol and cholesterol. At *p* < 0.05, we detected 93 plasma metabolites altered by fructose, with 62 metabolites increased and 31 decreased; in feces, 94 metabolites were altered by fructose, with 41 metabolites increased and 53 decreased. Ten metabolites overlapped between plasma and feces, including increased eicosenoylcarnitine, glutarate, and 1-(1-enyl-palmitoyl)-2-arachidonoyl-GPE (Figure 3A–C, Table S1), suggesting that some of them were potentially derived from gut microbiota metabolism. Pathway enrichment analysis of the differential metabolites affected by fructose consumption revealed alterations in fatty acid metabolism (acyl glycine), ceramides, and hexosylceramides in plasma, while in feces fatty acid metabolism (acyl choline), fatty acid metabolism (acyl carnitine), and primary bile acid metabolism were distinctly affected. Sterol pathway was altered in both plasma and feces of B6 mice (Figure 3A, Table S2).

To further prioritize metabolites altered by fructose consumption in B6 mice, we used four statistical and machine learning algorithms described in the previous section (Figure 2D,E, Table S5). No differential plasma metabolites were detected consistently across all methods, instead seven metabolites including ceramide (*N*-palmitoyl-sphingosine), hexosylceramide (glycosyl-*N*-palmitoyl-sphingosine), and cholesterol were detected across three statistical tests (Figure 3D). In feces, seven metabolites were detected consistently across methods, including three sulfated bile acids (chenodoxycholic acid sulfate (CDCA-S), cholate sulfate (CA-S), and taurocholenate sulfate) and three taurine-conjugated metabolites (succinoyltaurine, *N*-acetyltaurine, and taurocholenate sulfate). *N*,*N*,*N*-trimethyl-5-aminovalerate was also detected across all four methods, which has been reported to promote liver steatosis in mice consuming a high fat diet [26].

### 2.5. Correlation of Fructose-Responsive Metabolites with Fecal Microbiota and Metabolic Phenotypes in B6 Mice

In contrast to DBA mice, B6 mice showed no correlation between fructose-responsive metabolites and metabolic phenotypes such as adiposity and glucose intolerance, likely due to the limited phenotypic responses to fructose treatment (Table S4). We next performed correlation analysis between fructose responsive metabolites and microbes altered by fructose (decrease of *Dehalobacterium* and unknown genus of *Mogibacteriaceae*, and increased *Akkermansia*) [7]. Seven plasma metabolites in B6 mice (indolacetate, carotene diol, glycosyl-*N*-palmitoyl-sphingosine, cholesterol, *N*-palmitoyl-sphingosine, and sphingomyelin) that were increased by fructose consumption were correlated with all three bacteria (Table 2). In feces, 20 metabolites were correlated with one or more of the fructose-responsive fecal microbes. Eight of the 20 fecal metabolites were taurine-conjugated metabolite, which were all decreased by fructose, and six correlated with *Dehalobacterium* (Figure 3F,G, Table 3). We also noted that there were distinct correlations between bile acids and bacteria. Four primary bile acids (CDCA-S, CA-S, tauro-beta muricholate (Tβ-MCA), taurocholate) were all correlated with both *Dehalobacterium* and an unknown genus of *Magibacteriaceae*, while two secondary bile acids (taurochenodeoxycholic acid (7 or 24)-sulfate (TCDCA-S) and taurohyodeoxycholic acid (THDCA)), both taurine-conjugated, were negatively correlated with *Akkermansia* (Table 3). In B6 mice, none of the differential metabolites were correlated to both phenotypes and bacteria. Instead, most of the metabolites prioritized in Figure 3D,E were correlated with one or more fructose-responsive microbes (Figure 3H).

### 2.6. Comparison between DBA and B6 Reveals Strain-Specific Metabolic Pathways in Response to Fructose Consumption

**Figure 4:**
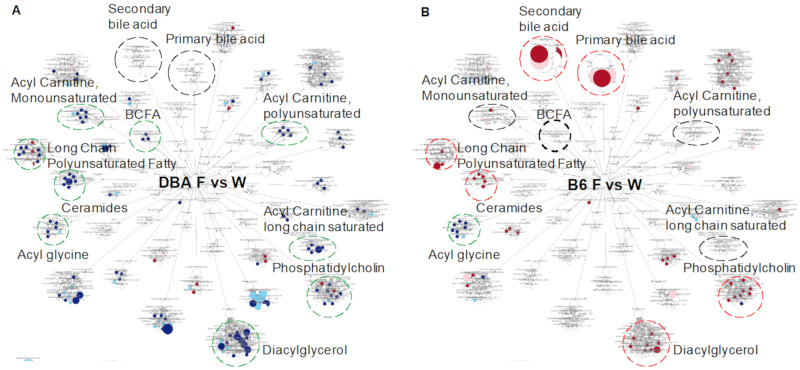
Plasma lipid pathways and metabolites altered by fructose consumption in DBA (**A**) and B6 mice (**B**). Dotted circles are lipid sub-pathways with contrasts between DBA and B6. Circles with red, green, black color indicate overall pathway level increase, decrease, or no change by fructose, respectively. Red and blue dots indicate lipid metabolites upregulated and downregulated at *p* < 0.05, respectively, in fructose-treated mice compared with controls. Pink and cyan dots indicate lipid metabolites upregulated and downregulated at 0.05 < *p* < 0.1, respectively. Dot size indicates the relative magnitude of change. *n* = 5–6/group. Statistical significance was determined by ANOVA contrast.

Our study indicates that both the baseline and fructose-responsive metabolomic profiles are dramatically different between B6 and DBA mice (Figure 1D–H, Figure 2A–C, Figure 3A–C). Since the metabolites categorized in the lipid pathway encompass nearly half (42–48%) of all detected metabolites (Figure 1B,C) and plasma metabolites are the collective outcome of host/gut microbiota metabolism, we further investigated lipid sub-pathways in plasma to compare the fructose response between DBA and B6 mice. Overall, more metabolites and pathways were decreased in DBA, while increased or unchanged in B6 mice by fructose. These metabolic pathways include BCFAs, ceramides, acyl carnitine, phosphatidylcholine, and diacylglycerol (Figure 4A,B). In contrast, primary and secondary bile acids were not altered in DBA mice but were significantly increased in B6 mice by fructose consumption. Only acyl glycine was consistently decreased in both mouse strains followed by fructose consumption. Overall, our results suggest distinct and somewhat opposite effects of fructose on lipid metabolism between DBA and B6 mice.

**Figure 5:**
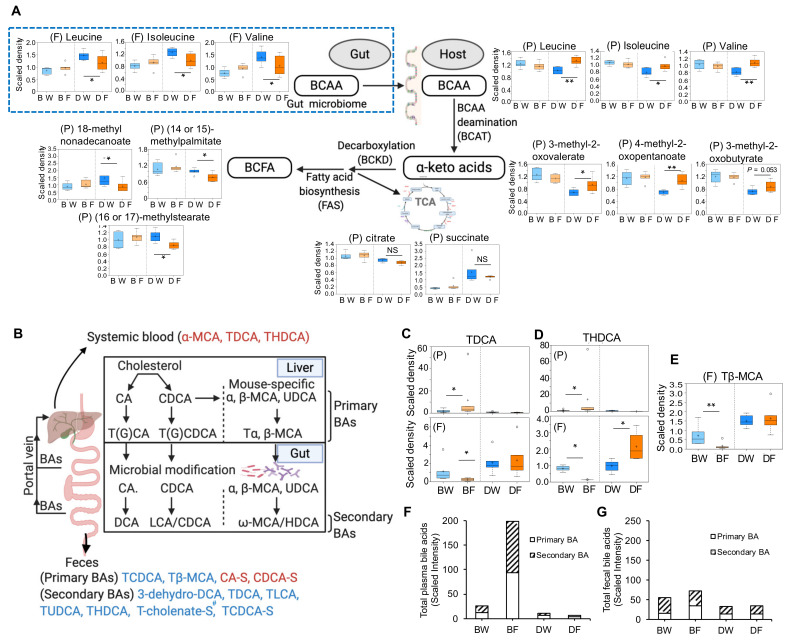
Strain-specific metabolic pathways altered by fructose consumption between DBA and B6 mice. (**A**) Alterations in BCAA and BCFA metabolism pathways in DBA mice. Box plots show the levels of relevant plasma or fecal metabolites in water and fructose groups of DBA mice. BCAA, branched chain amino acid; BCFA, branched chain fatty acid; BCAT, branched chain aminotransferase; BCKD, branched-chain α-ketoacid dehydrogenase complex; FAS, fatty acid synthase, TCA, tricarboxylic acid cycle. (**B**) Schematic representation of enterohepatic circulation of bile acids and differentially altered bile acids in the plasma or feces of B6 mice. Blue or red colored bile acids are the decreased or increased bile acids by fructose consumption in B6 mice, respectively. G, glycine-conjugated species; T, taurine-conjugated species; S, sulfated species; CA, cholic acid; CDCA, chenodeoxycholic acid; MCA, muricholic acids; UDCA, ursodeoxycholic acid; DCA, deoxy cholic acid; LCA, lithocholic acid; HDCA, hyodeoxycholic acid. (**C**–**E**) Box plots of select secondary (**C**,**D**) and primary (**E**) bile acids in plasma (upper panels of **C**,**D**) and feces (lower panels of **C**–**E**) of B6 and DBA treated by regular water or fructose water. Total bile acid in plasma (**F**) and feces (**G**). *n* = 5–6/group. Statistical significance was determined by ANOVA contrast (* *p* < 0.05, ** *p* < 0.01). NS indicates non-significant difference. BW, B6 water group; BF, B6 fructose group; DW, DBA water group; DF, DBA fructose group; P, plasma; F, feces. Symbol (#) next to metabolite indicates the compound has not been officially confirmed based on a standard, but confident in its identity.

High levels of BCAAs contribute to the development of obesity-associated insulin resistance [11] and BCAA catabolism is tightly linked to BCFA production [13]. In DBA mice, fructose treatment resulted in altered BCAA and BCFA metabolism (Figure 2A and Figure 4A) in feces and/or plasma, and BCFA metabolism alteration was top-ranked in plasma (Table S2). Upon fructose consumption, there was a significant decrease in BCAAs in feces (Figure 5A, Table S1). In plasma, BCAAs and their deaminated metabolites α-ketoacids (3-methyl-2-oxovalerate, 4-methyl-2-oxopentanoate, and 3-methyl-2-oxobutyrate) were significantly increased in response to fructose, without changes in the main catabolism intermediates in the tricarboxylic acid (TCA) cycle including citrate and succinate. However, all three metabolites (18-methylnonadecanoate, (14 or 15)-methylpalmitate, and (16 or 17)-methylstearate) detected in the BCFA pathway were significantly decreased by fructose (*p* < 0.05) (Figure 5A). These results suggest that fructose consumption inhibits BCFA synthesis from α-ketoacids, which in turn leads to BCAA/α-ketoacids accumulation and BCFA reduction in the plasma (Figure 5A). Compared to DBA mice, B6 mice showed no changes in these metabolites by fructose. It is reported that obesity-related insulin resistance is associated positively with BCAA and negatively with BCFA [13,16]. High levels of BCAAs and low levels of BCFAs in DBA mice by fructose consumption as observed in the current study thus agree with the development of obesity and glucose intolerance in DBA but not in B6, as shown in our previous study [6]. 

In B6 mice, fructose consumption significantly altered primary and secondary bile acids in plasma and feces, which was not observed in DBA mice (Figure 4A,B, Table S2). The gut microbiota plays an important role in bile acid synthesis and modification, and the resulting primary or secondary bile acids modulate host metabolism through farnesoid X receptor (FXR) in the liver and ileum [27]. FXR activation inhibits bile acid synthesis and induces bile acid secretion into the small intestine [28]. Interestingly, the fructose-responsive bile acids in B6 feces were mostly taurine-conjugated primary bile acids [taurochenodeoxycholate (TCDCA), Tβ-MCA] and secondary bile acids [taurodeoxycholate (TDCA), taurolithocholate (TLCA), tauroursodeoxycholate (TUDCA), THDCA, taurocholenate sulfate, TCDCA-S], all of which were significantly decreased by fructose treatment and some of them (TCDCA-S and THDCA) were correlated with *Akkermansia* (Figure 5B, Table 3). However, salt-conjugated primary bile acids including CA-S and CDCA-S in feces were increased by fructose consumption and correlated with *Dehalobacterium* and unknown genus of *Magibacteriaceae* (Figure 5B, Table 3). In plasma, fructose consumption increased TDCA and THDCA, which were decreased in feces (Figure 5C,D). We also noted the significant decrease of fecal Tβ-MCA by fructose consumption (Figure 5E), which correlated with *Dehalobacterium* and unknown genus of *Magibacteriaceae* (Table 3). Tβ-MCA is reported as a potent FXR antagonist in the ilium and regulates bile acid biosynthesis [29]. When total bile acids were considered, plasma but not fecal bile acids were markedly increased upon fructose consumption in B6 mice (Figure 5F,G). Overall, the decreased Tβ-MCA may inhibit bile acid biosynthesis from liver cholesterol, which may lead to the increase in plasma cholesterol concentration as we observed in the current metabolomics data (Figure 3B) or previous biochemical test [6].

## 3. Discussion

Our previous studies showed that three mouse strains representing a range of genetic diversity differed in their metabolic, transcriptomic, and microbiome responses to high fructose treatment [6,7]. Here we investigated whether the disparate genetic background and microbiome shape the metabolomic profiles with or without fructose consumption to better understand the differential risks to metabolic syndrome between DBA (fructose-sensitive) and B6 (fructose-resistant) mice. Our global metabolomics analysis revealed major differences between mouse strains in both the baseline and fructose-responsive metabolomic profiles. In DBA mice, several metabolites including plasma 1-methylnicotinamide and fecal 1-methyl-5-imidazoleacetate, lysyl-leucine, and enterodiol were identified as consistent differential metabolites by fructose across multiple statistical methods, most of which were strongly associated with adiposity and/or fructose-responsive microbes including *Rikenellaceae*, *Proteobacteriacea*, and/or *Pseudomonas*. In B6 mice, salt-conjugated primary bile acids, taurine-conjugated bile acids, and *N*,*N*,*N*-trimethyl-5-aminovalerate were prioritized among the differential metabolites altered by fructose in feces, most of which were associated with fructose-responsive bacteria *Dehalobacterium*, *Magibacteriaceae*, and/or *Akkermansia*. We also observed distinctly altered BCAA/BCFA metabolism in DBA mice and bile acid metabolism in B6 mice by fructose consumption. These results support that mouse strain-specific metabolomic profiles in response to fructose are likely shaped by mouse strain-specific fructose metabolism, gut microbiome, and host tissues, and may serve as biomarkers or play a regulatory role in differential specificity of fructose-induced metabolic syndrome in genetically different mice.

The metabolome reflects the overall metabolic phenotype which is integrated by host genetics, microbiome activity, and nutrition (or environment), and therefore, the bidirectional interaction of these factors creates a host-specific metabolomic profile as the collective outcome of host/gut microbiota metabolism [30,31]. It is reported that ~10% of metabolites found in mammalian blood are derived from the gut microbiota [32]. We observed that baseline plasma and fecal metabolite profiles and their responses to fructose were markedly different between DBA and B6 mice, likely reflecting their genetic and microbiome differences.

In the plasma of DBA mice fed by fructose, most of the altered metabolic pathways (9 out of 11 altered pathways) were associated with lipid metabolism, which were downregulated. Among these, fatty acid metabolism of acyl carnitine (polyunsaturated, monounsaturated, and long chain saturated) was remarkably altered toward decrease (Figure 4A, Table S2), which is consistent with other studies reporting the lowered acyl carnitine level under high fructose treatment in human or in vitro [22,33]. We speculate the decreased acyl carnitine by high fructose consumption might be associated with β-oxidation suppression since a reduced β-oxidation of free fatty acids is observed in humans and rat model treated by high fructose [34,35]. 

In DBA feces, downregulation of amino acid pathways by fructose was prominent, which include glycine, serine and threonine metabolism, phenylalanine metabolism, histidine metabolism, and BCAA metabolism. The host- and gut microbiota-derived proteases and peptidases break down dietary protein to amino acids, which are absorbed to host, or reach to colon and further metabolized to SCFAs including acetate, butyrate, propionate and BCFAs by colonic microbes [14].

In response to fructose consumption in DBA mice, plasma BCAAs and BCAA aminotransferase (BCAT)-mediated catabolic metabolites, α-ketoacids were significantly increased with no changes of TCA cycle intermediary metabolites. On the other hand, BCFA metabolism was the most significantly decreased pathway by fructose in plasma (Figure 4A, Table S2). These results suggest fructose consumption may induce the accumulation of BCAAs and α-keto acids through inhibition of BCFAs production from α-keto acids or acyl CoAs. However, there was no distinct change of BCAA catabolic metabolites in feces (Figure S1), which suggests that the changes of plasma BCAAs/BCFAs were derived from host endogenous metabolism. High BCAAs and low BCFAs in plasma or adipose tissue have been reported to play a role in the development of obesity and/or obesity-associated insulin resistance [11,13,15,16]. BCFAs are produced during fermentation of amino acids including BCAAs in the intestine by microbiota such as Bacillus [36] as well as being synthesized in the host adipose tissue and muscle during BCAA catabolism [13]. Our observation supports that the altered BCAA/BCFA metabolism by fructose may partly contribute to the onset of obesity and glucose intolerance in DBA mice. 

Among the hundreds of fructose-responsive metabolites in DBA mice, we prioritize metabolites consistently significant across various statistical methods. One metabolite (1-methylnicotinamide) in plasma and nine metabolites in feces were among these from DBA mice. 1-methylnicotinamide is converted from nicotinamide, a bioactive form of nicotinic acid (vitamin B3) by microbial activity in the gut or host liver enzymes [NNMT (Nicotinamide *N*-methyltransferase)]. 1-methylnicotinamide is known as a potent anti-inflammatory biochemical [37] and increases in obesity, diabetes, and coronary artery disease [23,24]. In our study, 1-methylnicotinamide was increased by fructose and correlated with adiposity, which agrees with the development of obesity and glucose intolerance in fructose-treated DBA mice [6]. Fecal metabolites, 1-methyl-5-imidazoleacetate, lysyl-leucine, and several xenobiotics such as coumestrol were increased by fructose, and were correlated with host adiposity as well as one or more of the fructose-responsive microbes including unknown genus of *Rikenellaceae*, unknown genus of *Pseudomonadaceae*, and *Pseudomonas*. It has been reported that 1-methyl-5-imidazoleacetate in the proximal colon is associated with intestinal mucosal damage and microbial dysbiosis in a pig model [38]. Lysyl-leucine is a dipeptide composed of lysine and leucine, and is related with metabolic unwellness [39]. Coumesterol has anti-obesity effect through activation of brown adipose tissue [40]. These fructose-responsive metabolites in DBA mice may also interact with specific gut bacteria and play a role in fructose-induced metabolic dysregulation in this mouse strain. 

B6 mice, in contrast to DBA mice, had increased bile acids, long chain monounsaturated fatty acids, ceramides, diacylglycerol, and phosphatidylcholine pathways with no changes in acyl carnitine and BCFA pathways in plasma (Figure 4). These distinct metabolite profiles agree with the disparate responses between B6 and DBA after fructose consumption. The most distinct fructose-responsive pathways in B6 are related to bile acid metabolism in both plasma and feces, which were not observed in DBA mice (Figure 4 and Figure 5B, Table S2). Bile acid metabolism is orchestrated by gut microbiota and host metabolism and is related to cholesterol metabolism. In mice, bile acids including cholic acid, chenodeoxycholic acid, and MCAs (primary bile acids) are synthesized in the liver from cholesterol, and then conjugated with amino acid taurine. Upon food consumption (dietary lipids), conjugated primary bile acids are released into the duodenum, reabsorbed in the ileum, and recirculated in the liver, but those that escape reabsorption are deconjugated to deoxycholic acid and lithocholic acid (secondary bile acids) by colonic bacteria and reabsorbed through the portal system (Figure 5B) [27]. Overall, more than 95% of the bile acid pool is preserved through this recycling system. 

The gut microbiota plays an important role in bile acid biosynthesis and modification, and the resulting bile acids modulate host metabolism including glucose homeostasis, lipid and lipoprotein metabolism, energy expenditure through FXR and the G protein-coupled membrane receptor 5 (TGR5) [27,41]. In B6 mice feces, we observed a decrease in Tβ-MCA, a potent FXR antagonist, by fructose, which may trigger Fgf19 production to block bile acid biosynthesis from cholesterol in the liver [27]. This may increase liver cholesterol levels, leading to plasma cholesterol accumulation. Indeed, we observed alterations of plasma cholesterol level in B6 mice, with increased LDL, HDL, total cholesterol, and unesterified cholesterol [6]. We also observed significantly higher levels of individual taurine-conjugated bile acids (TDCA and THDCA) along with total bile acids in plasma of B6 mice with fructose consumption (Figure 5C,D). Increased total serum bile acids (especially taurine-conjugated bile acids) has been observed in inflamed liver or liver disease [42]. Fructose is known as a major mediator of nonalcoholic fatty liver disease [43], which may be related with the high level of total plasma bile acids, especially taurine-conjugated bile acids. It is known that bile acid metabolism can alter defecation [44]. However, we did not evaluate the form and frequency of defecation before and after fructose intake. Further experiments are warranted to confirm the relationship between elevated plasma bile acids and liver disease as well as defecation changes by fructose consumption in B6 mice. 

In B6 mice, we also prioritized seven metabolites in feces, which showed statistical robustness across various statistical tests. These include bile acids, *N*,*N*,*N*-trimethyl-5-aminovalera, and taurine conjugated metabolites. Most of them were correlated with one or more of the fructose-responsive bacteria. It is reported that intestinal microbes metabolize trimethyllysine to *N*,*N*,*N*-trimethyl-5-aminovalerate (increased by fructose), which reduces carnitine synthesis and hepatic fatty acid oxidation to promote liver steatosis in mouse model [26]. We noted that sulfate-conjugated primary bile acids were increased by fructose and they were negatively correlated with *Dehalobacterium* and *Magibacteriaceae* (decreased by fructose), which suggests that these microbes may be responsible for desulfation of primary bile acids. 

The prioritized differential metabolites in B6 mice also include some taurine-conjugated metabolites including succinoyltaurine and *N*-acetyltaurine (decrease by fructose). Taurine is a conditional amino acid in mice which comes from food or is synthesized in the liver. Taurine can form conjugates with several metabolites such as bile acids, gangliosides, fatty acids, acetic acids, etc. For bile acids, taurine conjugation enhances solubility, alleviates cytotoxicity, and promotes the formation of micelles in the bile and the facilitation of lipid absorption in the intestinal tract. Taurine has a wide range of cytoprotective effect and can be used as a target for the treatment of metabolic diseases such as diabetes, inflammatory diseases, and diseases of the muscle, the central nervous system, and the cardiovascular system [45,46]. We noted taurine and taurine-conjugated metabolites were decreased by fructose in B6 feces, suggesting decreased availability (Figure 5A and Figure S1). However, another study reported an increase in taurine level in feces of B6 mice fed high fructose diet [32], and the authors speculated that overspill of fructose reaches the colon, resulting in changes to the gut microbiota composition (especially fructophilic bacteria *Lactobacillus*), followed by increased bile acid deconjugation and taurine release. In our study, fructose did not seem to overspill to the colon (Figure S2) and fecal *Lactobacillus* level was not altered by fructose consumption in B6 mice [7], which suggests that gut microbiota composition can be associated with fructose availability, which in turn shapes metabolite profiles. Differences in the fructose intake route (fructose added to diet in the previous study and in drinking water in our study) and dosage may explain the discrepancy. 

Taken together, our results demonstrate that baseline metabolomic profiles differ and respond differentially to fructose between mice with different genetic background and gut microbiota, which may play a role in individualized risks to fructose-induced metabolic syndrome. We identified intriguing metabolites that may play a key role in determining the strain-specific metabolic phenotypes induced by fructose, which we will follow up in the future studies. However, we acknowledge that examining metabolomics and gut microbiome changes at one time point misses the dynamic changes in these biological entities. Future time course studies are warranted. Another limitation is that our study design does not allow the differentiation of direct vs. indirect effects of fructose on metabolites and gut microbiota. In addition, the relationship between metabolites and gut microbiota can be bidirectional, although changes in both can be viewed as the result of direct or indirect effect of fructose intake. Further experimental validation is warranted to explore the functional roles of the differential metabolites, such as 1-methyl-5-imidazoleacetate and Tβ-MCA, on the gut microbiota and metabolic phenotypes as well as the direct vs. indirect role of fructose intake on gut microbiome and metabolites.

## 4. Materials and Methods

### 4.1. Sample Collection

Plasma and fecal samples were collected from DBA and B6 mice after a 12-week fructose treatment, as previously described [6,7]. Briefly, seven-week-old male DBA and B6 mice (20–25 g) were obtained from the Jackson Laboratory (Bar Harber, ME, USA) and housed in a pathogen-free barrier facility at University of California, Los Angeles. Mice were fed Lab Rodent Diet 500 (LabDiet, St. Louis, MO, USA). After a one-week acclimation period, mice from each strain were randomly divided into two groups. One group was provided with regular water (control group, *n* = 6 mice/strain) and the other group was given 8% (*w*/*v*) fructose (3.75 kcal/g energy; NOW Real Food, Bloomingdale, IL, USA) dissolved in regular water (fructose group, *n* = 6 mice/strain) for 12 weeks ad libitum. In the end of the experiment, feces were collected at the beginning of the 12-h dark cycle (6 p.m.) and snap frozen, and then stored at −80 °C. Blood samples were collected through retro-orbital bleeding at 9 a.m. after a 12-h overnight fasting. Plasma and feces were shipped to Metabolon, Inc. (Durham, NC, USA), where samples were processed, as described previously [47] and global metabolites data were analyzed as shown in Figure 1A. All animal procedures were performed in accordance with the National Institutes of Health Guide for the Care and Use of Laboratory Animals. All experimental protocols were approved by the Institutional Animal Care and Use Committee at the University of California, Los Angeles, CA, USA, identification code is 2012-059.

### 4.2. Sample Preparation and LC-MS/MS Analysis

Non-targeted global metabolomic profiling in plasma and feces was performed at Metabolon, Inc. (Durham, NC, USA) [48,49,50]. From each mouse, 50 uL of plasma or 2 mg of lyophilized feces were used for analysis [51]. In brief, extraction of samples was performed using an automated liquid handling robot (Hamilton LabStar, Hamilton Robotics, Inc., Reno, NV, USA), where 100% methanol was added to each sample at a 5:1 ratio (250 uL methanol for 50 uL sample) to precipitate proteins. The resulting supernatant was divided to four aliquots, dried, and reconstituted in solvents compatible to each of the four methods; Each reconstitution solvent contained a series of standards at fixed concentrations to ensure injection and chromatographic consistency. The first aliquot was reconstituted using 50 μL of 0.1% formic acid in water (pH ~ 3.5) and analyzed using acidic positive ion conditions, chromatographically optimized for more hydrophilic compounds. In this method, the extract was gradient eluted from a C18 column using water and methanol, containing 0.05% perfluoropentanoic acid (PFPA) and 0.1% formic acid (FA). The second aliquot was reconstituted using 50 μL of 0.1% formic acid in water (pH ~ 3.5) and analyzed using acidic positive ion conditions, however it was chromatographically optimized for more hydrophobic compounds. In this method, the extract was gradient eluted from the same aforementioned C18 column using methanol, acetonitrile, water, 0.05% PFPA, and 0.01% FA and was operated at an overall higher organic content. The third aliquot was reconstituted using 50 μL of 6.5 mM ammonium bicarbonate in water (pH 8) and analyzed using basic negative ion optimized conditions using a separate dedicated C18 column. The basic extracts were gradient eluted from the column using methanol and water, however with 6.5 mM Ammonium Bicarbonate at pH 8. The fourth aliquot was reconstituted using 50 μL of 6.5 mM ammonium bicarbonate in water (pH 8) and analyzed via negative ionization following elution from a HILIC column (Waters UPLC BEH Amide 2.1 × 150 mm, 1.7 µm) using a gradient consisting of water and acetonitrile with 10 mM Ammonium Formate, pH 10.8. All methods utilized a Waters ACQUITY UPLC (Milford, MA, USA) and a Q-Exactive high resolution/accurate mass spectrometer (Thermo Scientific, San Jose, CA, USA) with a heated electrospray ionization (HESI-II) source and Orbitrap mass analyzer (Thermo Scientific, San Jose, CA, USA) operated at 35,000 mass resolution.

### 4.3. Data Extraction and Metabolites Identification

Raw data was extracted, peak-identified and quality control (QC) processed using Metabolon’s hardware and software. Briefly, metabolites were identified by comparison to library entries of purified standards. Metabolon maintains a library based on authenticated standards that contains the retention time/index (RI), mass to charge ratio (*m*/*z*), and chromatographic data (including MS/MS spectral data) on all molecules present in the library. Furthermore, biochemical identifications are based on three criteria: retention index within a narrow RI window of the proposed identification, accurate mass match to the library +/− 10 ppm, and the MS/MS forward and reverse scores between the experimental data and authentic standards. A variety of curation procedures were carried out to ensure that a high-quality data set was made available for statistical analysis and data interpretation. The QC and curation processes were designed to ensure accurate and consistent identification of true chemical entities, and to remove those representing system artifacts, mis-assignments, and background noise.

### 4.4. Metabolites Quantification and Data Normalization

Peaks were quantified using area under the curve (AUC). A data normalization step was performed to correct variation resulting from instrument inter-day tuning differences. Essentially, each compound was corrected in run-day blocks by registering the medians to equal one and normalizing each data point proportionately. Missing values are imputed with minimum observed values for each compound.

### 4.5. Principal Component Analysis and Differential Metabolites

Both analyses were performed at Metabolon, Inc. (Durham, NC, USA). PCA was used to visualize variance distribution. We noted the presence of an outlier (pointed by the arrow in Figure 1E) among B6 fecal samples treated by fructose and excluded it from the data set for the downstream analysis. Differential metabolites analyses were performed using ArrayStudio software (OmicSoft Corp., St. Morrisville, NC, USA) on log transformed data. ANOVA contrasts were used to determine the metabolites that significantly differed between experimental groups (fructose vs. water group, DBA vs. B6 mice). False discovery rate (*q*-value) was calculated to take into account the multiple comparisons that normally occur in metabolomics studies, with *q* < 0.05 used as an indication of significance with high confidence. In cases where significant metabolites at *q* < 0.05 were not enough to run downstream pathway analysis such as in the analysis of differential metabolites by fructose in DBA and B6 mice, *p* < 0.05 was used to select differential metabolites.

### 4.6. Metabolic Pathway Analysis of Significantly Altered Metabolites

Pathway enrichment analysis of the significantly altered metabolites in plasma or feces were performed within MetaboLync pathway analysis (MPA) software (Metabolon, Inc.), and the enrichment score was calculated by the following equation: [# of significant metabolites in pathway (k)/total # of detected metabolites in pathway (m)]/[total # of significant metabolites (*n*)/total # of detected metabolites (N)]. The statistical significance was calculated using a hypergeometric test. The top representative metabolic pathways were selected with an enrichment score greater than 1.5 and a hypergeometric *p*-value < 0.05. 

### 4.7. Prioritization of Statistically Robust Differential Metabolites

To prioritize fructose-responsive metabolites, we identified consistently significant metabolites across four statistical methods: *t*-test/fold change, Significance analysis of microarrays (SAM), Partial least squares discriminant analysis (PLS-DA), and random forest in the MetaboAnalyst [https://www.metaboanalyst.ca/ (accessed on 12 March 2020)] [52]. In each analysis, significant metabolites were identified using a *p*-value threshold of 0.05 and fold change threshold of 2 for *t*-test/fold change-based selection, FDR (*q*-value) threshold of 0.05 for SAM-based analysis, top 30 compounds ranked by VIP scores at PLS-DA model, top 30 metabolites by feature importance measures at Random forest approach. Four sets of significant metabolites were overlapped in Venn diagrams (InteractiVenn tool, http://www.interactivenn.net, accessed on 12 September 2020).

### 4.8. Correlation Analysis

Correlation between fructose-responsive metabolites (*q* < 0.05) in plasma or feces and fructose-responsive gut microbiota as well as metabolic phenotypes including body weight, AUC of glucose tolerance, lean mass, adiposity, plasma triglyceride, free fatty acid, and plasma insulin was assessed using Biweight midcorrelation (bicor) [53]. Statistical *p*-values were adjusted using the Benjamini-Hochberg approach and FDR < 0.05 was considered significant. Analysis was done in R.

## 5. Conclusions

Our multi-omics (metabolome, gut microbiome, phenome) integrative studies of two mouse strains established a strain-specific host-metabolome interaction orchestrated with the gut microbiome in fructose-induced metabolic syndrome. Our results demonstrate that metabolomic profiles differ between mouse strains with different genetic background and microbial composition. We also showed that alterations in the metabolomic profiles in response to fructose is mouse strain-specific, with BCAAs/BCFAs prominent in DBA and bile acids specific to B6 mice, and that these metabolites are associated with different sets of microbiota and strain-specific disease phenotypes. Interplay between the metabolome, genetics, and microbiome likely determine the individualized risks to fructose-induced metabolic syndrome. Further studies are required to confirm the causal relationships between the prioritized metabolites, microbiota, and strain-specific phenotypes.

## Figures and Tables

**Table 1:** Correlation analysis between fructose-responsive fecal microbiota and metabolites in plasma and feces of DBA mice.

				Fold Change ^2^	*Rikenellaceae* (Unknown)		*Pseudomonadaceae* (Unknown)		*Pseudomonas*	
Metabolite	Sample ^1^	Pathway	Subpathway		Corr ^3^	FDR ^4^	Corr	FDR	Corr	FDR
1,5-anhydroglucitol (1,5-AG)	Plasma	Carbohydrate	Glycolysis, Gluconeogenesis, and Pyruvate Metabolism	1.32 *	−0.91	0.007				
1-oleoyl-2-arachidonoyl-GPI (18:1/20:4) ^#^	Plasma	Lipid	Phosphatidylinositol (Pl)	1.45 *			0.73	0.034		
1-methyl-5-imidazoleacetate	Feces	Amino Acid	Histidine Metabolism	2.03 *	−0.68	0.037	0.52	0.099	0.72	0.035
4-hydroxyphenylacetate	Feces	Amino Acid	Phenylalanine Metabolism	2.84 **	−0.64	0.046	0.70	0.035	0.71	0.035
protoporphyrin IX	Feces	Cofactors and Vitamins	Hemoglobin and Porphyrin Metabolism	0.24 **	0.64	0.045	−0.64	0.045		
behenoyl ethanolamide (22:0) ^#^	Feces	Lipid	Endocannabinoid	0.49 *	0.79	0.019	−0.67	0.039	−0.65	0.045
lignoceroyl ethanolamide (24:0) ^#^	Feces	Lipid	Endocannabinoid	0.50 *	0.73	0.035	−0.68	0.037	−0.59	0.063
2-hydroxyarachidate ^#^	Feces	Lipid	Fatty Acid, Monohydroxy	0.34 **	0.63	0.045	−0.71	0.035	−0.70	0.035
2-hydroxybehenate	Feces	Lipid	Fatty Acid, Monohydroxy	0.29 **	0.67	0.039	−0.71	0.035	−0.70	0.035
2-hydroxylignocerate ^#^	Feces	Lipid	Fatty Acid, Monohydroxy	0.41 **	0.64	0.045	−0.85	0.010	−0.69	0.035
2-hydroxynervonate ^#^	Feces	Lipid	Fatty Acid, Monohydroxy	0.46 **	0.72	0.035	−0.76	0.026	−0.64	0.045
choline	Feces	Lipid	Phospholipid Metabolism	1.54 *	−0.89	0.006				
taurohyodeoxycholic acid	Feces	Lipid	Secondary Bile Acid Metabolism	2.20 **			0.80	0.02		
cholesterol	Feces	Lipid	Sterol	0.67 *	0.78	0.019			−0.75	0.028
sphinganine	Feces	Lipid	Sphingolipid Synthesis	0.53 *	0.69	0.036	−0.68	0.037	−0.63	0.045
lysylleucine	Feces	Peptide	Dipeptide	3.24 *			0.86	0.010	0.69	0.035
apigenin	Feces	Xenobiotics	Food Component/Plant	2.22 *	−0.78	0.019				
coumestrol	Feces	Xenobiotics	Food Component/Plant	3.00 **	−0.78	0.020				
enterodiol	Feces	Xenobiotics	Food Component/Plant	2.06 *	−0.84	0.011			0.62	0.048
enterolactone	Feces	Xenobiotics	Food Component/Plant	4.04 **	−0.66	0.039				
pinosylvin	Feces	Xenobiotics	Food Component/Plant	6.36 *	−0.66	0.039				

^#^ Indicates compounds that have not been officially confirmed based on a standard, but confident in its identity. ^1^ Sample size *n* = 5–6/group. ^2^ Fold change is the metabolites ratio between fructose and water groups (fructose/water). * *p*-value < 0.05, ** *p*-value < 0.01. ^3^ Bicorr correlation coefficient. ^4^ False discovery rate.

**Table 2:** Correlation analysis between fructose-responsive plasma metabolites and fecal microbiota in B6 mice.

Fold Change ^2^					*Dehalobacterium*		*Magibacteriaceae* (Unknown)		*Akkermansia*	
Metabolite	Sample ^1^	Pathway	Subpathway		Corr ^3^	FDR ^4^	Corr	FDR	Corr	FDR
Indoleacetate	Plasma	Amino Acid	Tryptophan Metabolism	1.78 **	−0.83	0.004	−0.85	0.003	0.64	0.041
Carotene diol	Plasma	Cofactors/Vitamins	Vitamin A Metabolism	1.69 *	−0.79	0.008	−0.77	0.010	0.88	0.002
Glycosyl-*N*-palmitoyl-sphingosine (d18:1/16:0)	Plasma	Lipid	Hexosylceramides	1.42 **	−0.77	0.010	−0.76	0.010	0.83	0.004
Cholesterol	Plasma	Lipid	Sterol	1.30 *	−0.63	0.041	−0.64	0.041	0.64	0.041
*N*-palmitoyl-sphingosine (d18:1/16:0)	Plasma	Lipid	Ceramides	1.63 **	−0.76	0.001	−0.85	0.003	0.73	0.014
Sphingomyelin (d18:1/14:0, d16:1/16:0)	Plasma	Lipid	Sphingomyelins	1.44 *	−0.89	0.002	−0.84	0.003	0.94	0.001
Sphingomyelin (d17:1/16:0, d18:1/15:0, d16:1/17:0)	Plasma	Lipid	Sphingomyelins	1.38 *	−0.88	0.002	-0.78	0.010	0.90	0.002

^1^ Sample size *n* = 5–6/group. ^2^ Fold change is the metabolites ratio between fructose and water groups (fructose/water). * *p*-value < 0.05, ** *p*-value < 0.01. ^3^ Bicorr correlation coefficient. ^4^ False discovery rate.

**Table 3:** Correlation analysis between fructose-responsive fecal metabolites and fecal microbiota in B6 mice.

				Fold Change ^2^	*Dehalobacterium*		*Magibacteriaceae* (Unknown)		*Akkermansia*	
Metabolite	Sample ^1^	Pathway	Subpathway		Corr ^3^	FDR ^4^	Corr	Adjp	Corr	Adjp
Isobutyrylglycine (C4)	Feces	Amino Acid	Leucine, Isoleucine and Valine	3.32 *	−0.77	0.033				
*N*,*N*-dimethyl-5-aminovalerate	Feces	Amino Acid	Lysine Metabolism	1.54 *	−0.75	0.038			0.77	0.036
*N*-acetyltaurine	Feces	Amino Acid	Methionine, Cysteine, SAM and Taurine Metabolism	0.21 *	0.87	0.013	0.89	0.008		
Succinoyltaurine	Feces	Amino Acid	Methionine, Cysteine, SAM and Taurine Metabolism	0.31 *	0.88	0.009	0.94	0.002		
Taurine	Feces	Amino Acid	Methionine, Cysteine, SAM and Taurine Metabolism	0.34 *			0.85	0.017	−0.80	0.026
Biotin	Feces	Cofactors/ Vitamins	Biotin Metabolism	0.19 **			0.81	0.025		
Phenylacetyltaurine	Feces	Lipid	Acetylated Peptides	0.40 *	0.79	0.027	0.92	0.004	−0.78	0.032
*N*-linoleoyltaurine	Feces	Lipid	Endocannabinoid	0.34 *					−0.73	0.045
Stearoyl ethanolamide	Feces	Lipid	Endocannabinoid	2.07 *	−0.74	0.045	−0.83	0.022		
Lactosyl-*N*-palmitoyl-sphingosine (d18:1/16:0)	Feces	Lipid	Lactosylceramides	0.45 *	0.95	0.002	0.79	0.027		
Chenodeoxycholic acid- sulfate	Feces	Lipid	Primary Bile Acid	8.61 **	−0.80	0.025	−0.74	0.045		
Cholate Sulfate	Feces	Lipid	Primary Bile Acid	8.45 **	−0.84	0.017	−0.75	0.038		
Tauro-beta muricholate	Feces	Lipid	Primary Bile Acid	0.11 **	0.88	0.010	0.81	0.025		
Taurocholate	Feces	Lipid	Primary Bile Acid	0.34 *	0.77	0.036	0.76	0.036		
Taurochenodeoxycholic acid (7 or 24)-sulfate	Feces	Lipid	Secondary Bile Acid	0.00 **					−0.78	0.032
Taurohyodeoxycholic acid	Feces	Lipid	Secondary Bile Acid	0.16 **	0.82	0.024			−0.82	0.025
Beta-sitosterol	Feces	Lipid	Sterol	0.66 *	0.91	0.004	0.81	0.025		
Campesterol	Feces	Lipid	Sterol	0.64 *	0.92	0.004	0.82	0.025		
Cholesterol	Feces	Lipid	Sterol	0.70 *	0.76	0.037			−0.76	0.036
Stigmasterol	Feces	Lipid	Sterol	0.66 *	0.92	0.004	0.85	0.017		

^1^ Sample size *n* = 5–6/group. ^2^ Fold change is the metabolites ratio between fructose and water groups (fructose/water). * *p*-value < 0.05, ** *p*-value < 0.01. ^3^ Bicorr correlation coefficient. ^4^ False discovery rate.

## Data Availability

Detailed results are in Supplementary Materials. Raw data is available upon request.

## Supplementary Materials

The following are available online at https://www.mdpi.com/article/10.3390/metabo11060342/s1, Figure S1: BCAA catabolism-related metabolites in feces of DBA and B6 mice, Figure S2: Box plots of fructose and taurine in feces of DBA and B6 mice, Table S1: Differentially abundant metabolites between two groups in plasma and feces samples of DBA and B6 mice, Table S2: Representative metabolic pathways of the significantly different metabolites between two groups, Table S3: Identification of significant features between fructose group and water group in plasma and feces of DBA mice using four different statistical analysis (*t*-test/Fold change, SAM. PLS-DA, Random forest), Table S4: Correlation analysis between metabolites and metabolic phenotypes in plasma and feces of DBA mice, Table S5: Identification of significant features between fructose group and water group in plasma and feces of B6 mice using four different statistical analysis (*t*-test/Fold change, SAM. PLS-DA, Random forest).
