# Supplementary material for: Disparate Metabolomic Responses to Fructose Consumption between Different Mouse Strains and the Role of Gut Microbiota
Source: Metabolites. 2021 May 26;11(6):342. doi: 10.3390/metabo11060342 (PMC8228112; doi:10.3390/metabo11060342)
Supplement: Supplementary file 1 [file metabolites-11-00342-s001.zip › metabolites-1146974-supple/Ahn_Supplementary Figure .pdf]

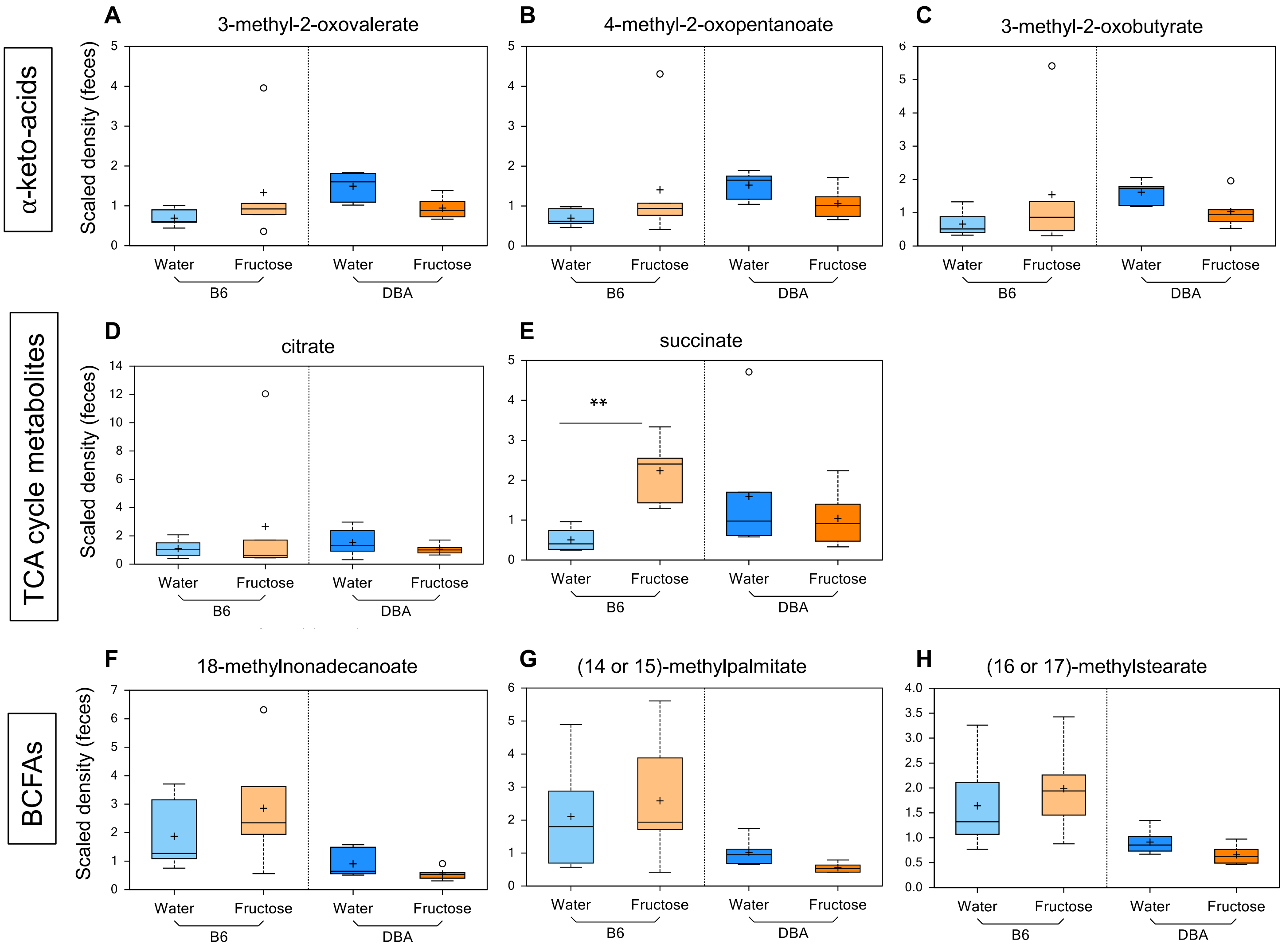

Figure S1. BCAA catabolism-related metabolites in feces of DBA and B6 mice.

n = 5-6/group. Statistical significance was determined by ANOVA contrast (*P < 0.05).

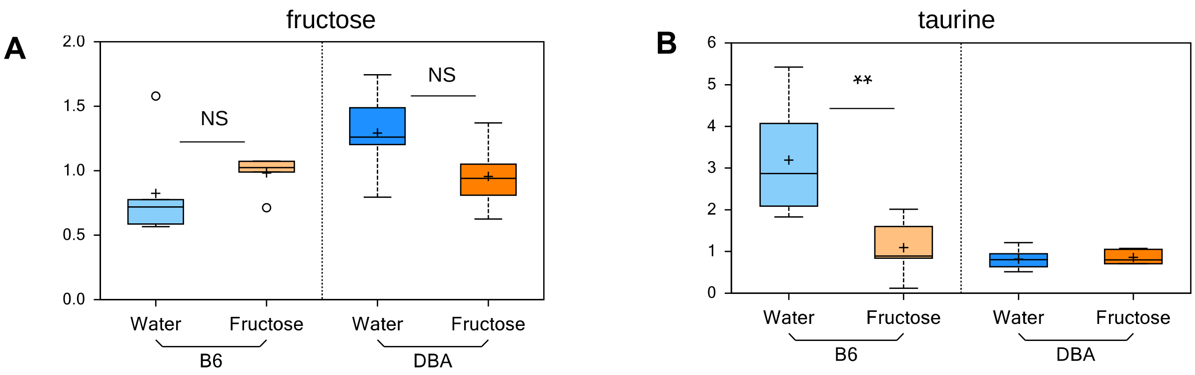

Figure S2. Box plots of fructose and taurine levels in feces of DBA and B6 mice.

n = 5-6/group. Statistical significance was determined by ANOVA contrast (**$P < 0.01$).

NS indicates non-significant differences.
